# Effects of Ranibizumab and Aflibercept on Human Müller Cells and Photoreceptors under Stress Conditions

**DOI:** 10.3390/ijms18030533

**Published:** 2017-03-01

**Authors:** Weiyong Shen, Belinda Yau, So-Ra Lee, Ling Zhu, Michelle Yam, Mark C. Gillies

**Affiliations:** Macula Research Group, Clinical Ophthalmology and Eye Health, Save Sight Institute, The University of Sydney, Sydney, NSW 2000, Australia; belinda.yau@sydney.edu.au (B.Y.); sora.lee@sydney.edu.au (S.-R.L.); ling.zhu@sydney.edu.au (L.Z.); michelle.yam@sydney.edu.au (M.Y.); mark.gillies@sydney.edu.au (M.C.G.)

**Keywords:** vascular endothelial growth factor, aflibercept, ranibizumab, Müller cells, photoreceptors, neurotrophic factors, heat shock proteins, redox proteins, interphotoreceptor retinoid-binding protein

## Abstract

Anti-vascular endothelial growth factor (VEGF) therapy has revolutionized the treatment of retinal vascular diseases. However, constitutive VEGF also acts as a trophic factor on retinal non-vascular cells. We have studied the effects of aflibercept and ranibizumab on human Müller cells and photoreceptors exposed to starvation media containing various concentrations of glucose, with or without CoCl_2_-induced hypoxia. Cell survival was assessed by calcein-AM cell viability assays. Expression of heat shock proteins (Hsp) and redox proteins thioredoxin 1 and 2 (TRX1, TRX2) was studied by Western blots. The production of neurotrophic factors in Müller cells and interphotoreceptor retinoid-binding protein (IRBP) in photoreceptors was measured by enzyme-linked immunosorbent assays. Aflibercept and ranibizumab did not affect the viability of both types of cells. Neither aflibercept nor ranibizumab affected the production of neurotrophic factors or expression of Hsp60 and Hsp90 in Müller cells. However, aflibercept but not ranibizumab affected the expression of Hsp60, Hsp9, TRX1 and TRX2 in photoreceptors. Aflibercept and ranibizumab both inhibited the production of IRBP in photoreceptors, aflibercept more so than ranibizumab. Our data indicates that the potential influence of aflibercept and ranibizumab on photoreceptors should be specifically monitored in clinical studies.

## 1. Introduction

The functional integrity of the retinal vasculature is important for eye health. Neovascular age-related macular degeneration (AMD) and diabetic retinopathy are characterized by vascular leak and growth of abnormal blood vessels [1,2]. Disruption of the balance between angiogenic stimulators and inhibitors in pathological conditions can lead to vascular leak and growth of new blood vessels [1].

AMD is the leading cause of irreversible blindness among people aged ≥65 years in the developed world. Neovascular AMD comprises only 10%–15% of all AMD cases but it accounts for >80% of severe vision loss or legal blindness resulting from the disease [3]. Diabetic retinopathy is widely considered to be a neurovascular disease. Diabetic macular edema occurs when the blood retinal barrier is disrupted, causing vascular leak and accumulation of extracellular fluid and protein deposits [4]. Despite their different etiologies, vision loss in AMD and diabetic retinopathy is ultimately caused by neuronal degeneration. Vascular leak and neovascularization may exacerbate the underlying neuronal damage, thus contributing to vision loss.

Vascular endothelial growth factor (VEGF) is a trophic endothelial growth factor that promotes endothelial migration, proliferation, survival and differentiation [1]. Overexpression of VEGF promotes vascular leak and ocular neovascularization. The advent of anti-VEGF agents such as ranibizumab (Lucentis) and aflibercept (Eylea) has revolutionized the treatment of retinal vascular diseases. Ranibizumab is a fully humanized small chain fragment that binds with high affinity to human VEGF-A, thereby preventing binding of VEGF-A to its receptors VEGFR-1 and VEGFR-2. Intravitreal injections of ranibizumab effectively reduce vascular leak and inhibit ocular neovascularization in clinical studies [1]. Aflibercept is a soluble VEGF decoy receptor that specifically recognizes and binds to VEGF-A, -B and placental growth factor (PLGF) [1,5]. Aflibercept is the newest anti-VEGF agent to be approved for treatment of neovascular AMD [1,5].

A growing body of evidence indicates that constitutive VEGF also acts as a trophic factor on non-vascular cells and plays an important role in the maintenance and function of retinal ganglion cells, Müller cells, photoreceptors, the retinal pigment epithelium (RPE) and choriocapillaries [6,7,8,9,10,11,12,13,14]. Therefore, chronic inhibition of VEGF may cause unwanted adverse effects on these retinal and choroidal cells.

Müller cells are the major type of glial cells in the retina. Ensheathing all retinal neurons including photoreceptor cells, Müller cells protect retinal neurons via release of neurotrophins and the antioxidant glutathione [15]. Müller cells have been reported to release neurotrophic factors such as neurotropins, including brain-derived neurotrophic factor (BDNF), neurotrophins 3 and 4 (NT3 and NT4) and pigment epithelium derived factor (PEDF), contributing to the health of photoreceptors and other neurons in the retina [16,17,18,19].

A number of studies have recently tested the safety profiles of ranibizumab [20,21,22,23] and aflibercept [20,23] using human RPE cells [20,21,22,23], rat ganglion cell-like/neuronal progenitor cells [23] and mouse 661w photoreceptor cells [23]. However, currently there is little information on the effects of anti-VEGF therapy on human Müller cells and photoreceptors in vitro. In this study, we assessed the effects of clinical doses of ranibizumab and aflibercept on cell survival and differential expression of cell stress markers by human MIO-M1 Müller cells and Y79 photoreceptors under various stressed conditions. The effects of aflibercept and ranibizumab on the production of neurotrophic factors in Müller cells and interphotoreceptor retinoid-binding protein (IRBP) in photoreceptors were also evaluated.

## 2. Results

### 2.1. Effects of Hyperglycemia and Hypoxia on the Survival of Müller Cells and Photoreceptors

**Figure 1:**
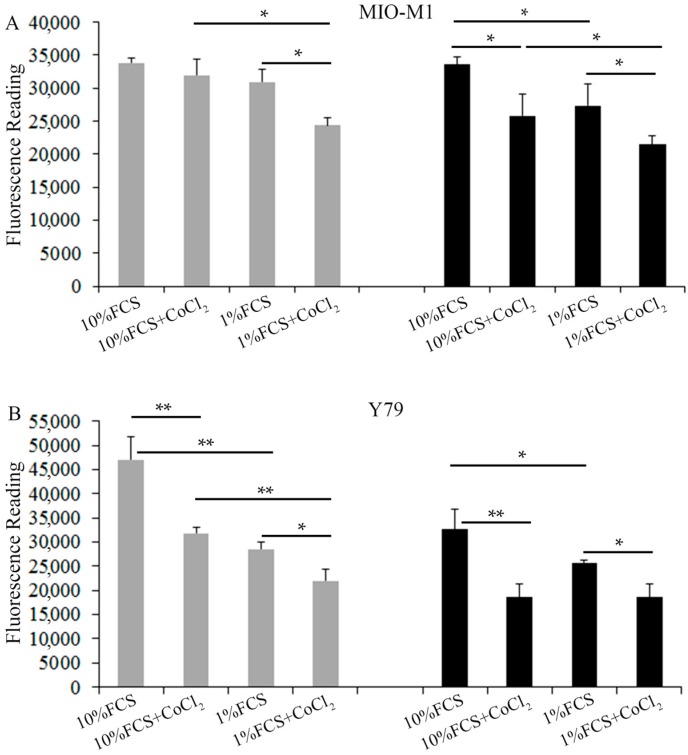
Effects of serum starvation, high glucose and hypoxia on cell survival of Müller cells and photoreceptors. Cell survival was measured by calcein-AM cell viability assays 24 h after incubating cells in test media. (**A**) Müller cell viability assays. Grey bars: 5 mM glucose. Black bars: 25 mM glucose. * *p* < 0.05, *n* = 6/group; (**B**) Y79 cell viability assays. Grey bars: 11 mM glucose. Black bars: 25 mM glucose. * *p* < 0.05 and ** *p* < 0.01 respectively, *n* = 6/group. FCS, fetal calf serum.

We compared Müller cells exposed to various stressed conditions with those cultured in medium containing 10% fetal calf serum (FCS) + low glucose (LG, 5 mM). We found that stress conditions consisting of 10% FCS + LG + CoCl_2_, 1% FCS + LG and 10% FCS + high glucose (HG, 25 mM) did not significantly affect the viability of Müller cells (Figure 1A). However, the combination of 1% FCS starvation with CoCl_2_-induced hypoxia or HG significantly reduced the Müller cell viability (Figure 1A). In Y79 photoreceptors, significant reduction of cell viability was observed when photoreceptors were incubated under stressed conditions including 10% FCS + CoCl_2_, 1% FCS starvation alone or 1% FCS starvation + CoCl_2_, regardless of the concentration of glucose (Figure 1B). High glucose (25 mM, HG) also significantly reduced the viability of Y79 cells compared with cells incubated in RPMI medium containing 10% FCS and 11 mM glucose (LG) (Figure 1B).

### 2.2. Effects of Cell Stress on Vascular Endothelial Growth Factor (VEGF)-A Production and Hypoxia-Inducible Factor 1α (HIF1α) Expression in Müller Cells and Photoreceptors

**Figure 2:**
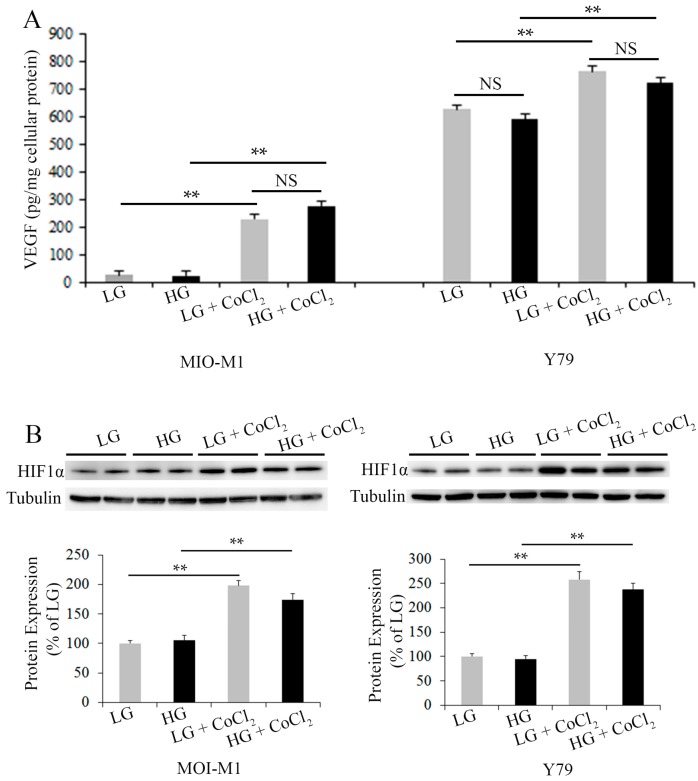
Effects of cell stress on VEGF-A and HIF1α expression in Müller cells and Y79 photoreceptors. (**A**) VEGF-A was measured by ELISA using conditioned media collected from MIO-M1 and Y79 cells, *n* = 4–5/group in Müller cells and *n* = 8/group in Y79 photoreceptors, ** *p* < 0.01; (**B**) Western blots for HIF1α using cellular proteins, *n* = 4/group, ** *p* < 0.01. For MIO-M1 Müller cells: Grey bars: 5 mM glucose. Black bars: 25 mM glucose. For Y79 photoreceptors: Grey bars: 11 mM glucose. Black bars: 25 mM glucose. NS, not significant; LG, low glucose; HG, high glucose.

In order to study the effects of stress on VEGF production by Müller cells and photoreceptors, we collected conditioned media for VEGF ELISA 24 h after culturing both types of cells in starvation media containing 1% FCS and various concentrations of glucose with and without CoCl_2_-induced hypoxia (Figure 2A). As expected, CoCl_2_-induced hypoxia significantly increased VEGF production in Müller cells and photoreceptors compared with the corresponding groups without hypoxia (Figure 2A). However, HG appeared to have little effect on the production of VEGF in both types of cells when compared with the corresponding groups cultured in media containing low glucose (Figure 2A).

As HIF1α regulates VEGF expression, we next performed Western blots to study changes in HIF1α expression in Müller cells and Y79 photoreceptors after exposure to starvation media containing 1% FCS and various concentrations of glucose, with and without CoCl_2_-induced hypoxia (Figure 2B). Consistent with the results of VEGF production, CoCl_2_-induced hypoxia significantly increased HIF1α expression in both types of retinal cells compared with the corresponding groups without hypoxia, while HG had little effect on HIF1α expression when compared with the corresponding groups cultured in LG media (Figure 2B).

### 2.3. Effects of Aflibercept and Ranibizumab on Müller Cell Survival

**Figure 3:**
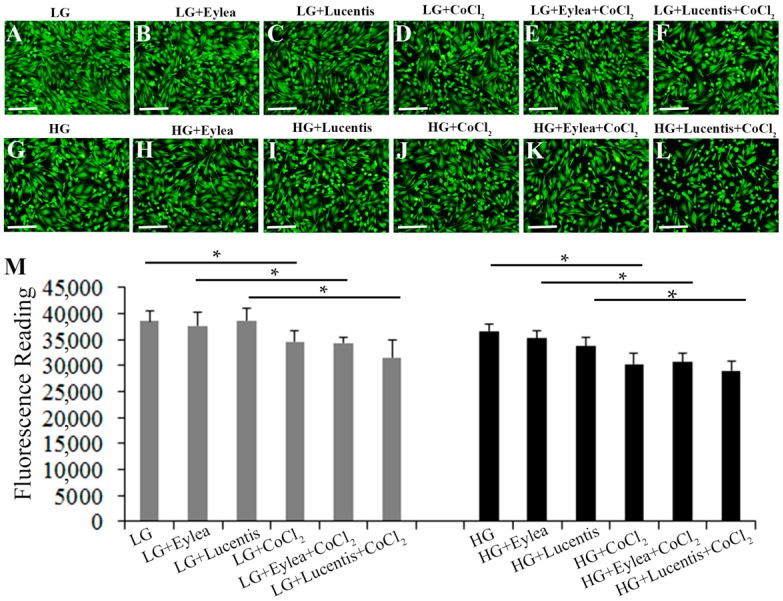
Aflibercept (Eylea, 0.5 mg/mL) and ranibizumab (Lucentis, 0.125 mg/mL) did not affect Müller cell survival under stress conditions. (**A**–**L**) Fluorescence images of calcein-AM-stained Müller cells exposed to stress media for 24 h. Scale bars: 50 µm; (**M**) Quantitative analysis of Müller cell viability by measuring fluorescence intensity after staining Müller cells with calcein-AM. * *p* < 0.05, *n* = 6/group.

After establishing the in vitro cell stress model in MIO-M1 Müller cells, we next tested the effects of anti-VEGF therapy on cell viability 24 h after incubating Müller cells in various stress media containing clinical doses of aflibercept (Eylea, 0.5 mg/mL) and ranibizumab (Lucentis, 0.125 mg/mL). Fluorescence microscopy of Müller cells stained with calcein-AM did not reveal changes in Müller cell morphology under the tested stress conditions (Figure 3A–L). Measurements of fluorescence intensity after calcein-AM staining indicated that CoCl_2_-induced hypoxia significantly reduced the Müller cell viability (Figure 3M). However, treatment with ranibizumab and aflibercept did not affect Müller cell survival when compared with each corresponding group cultured in starvation media containing various concentrations of glucose, with or without CoCl_2_-induced hypoxia (Figure 3M).

### 2.4. Effects of Aflibercept and Ranibizumab on Photoreceptor Cell Viability

**Figure 4:**
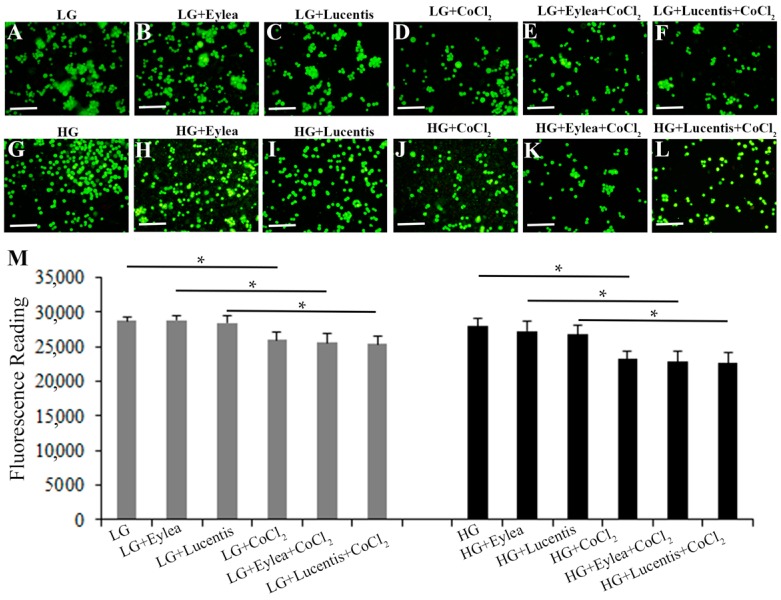
Aflibercept and ranibizumab did not affect Y79 photoreceptor cell survival under stress conditions. (**A**–**L**) Fluorescence images of calcein-AM-stained Y79 cells exposed to stress media containing LG (11 mM) or HG (25 mM) for 24 h. Scale bars: 50 µm; (**M**) Quantitative analysis of Y79 cell viability by measuring the fluorescence intensity after staining Y79 cells with calcein-AM. * *p* < 0.05, *n* = 6/group.

Fluorescence microscopy of Y79 photoreceptors stained with calcein-AM indicated that addition of aflibercept and ranibizumab into starvation media containing LG or HG did not affect Y79 cell survival but CoCl_2_-induced hypoxia reduced the cell viability (Figure 4A–L). This observation was further confirmed by quantitative measurement of fluorescence intensity after staining Y79 photoreceptors with calcein-AM (Figure 4M).

### 2.5. Effects of Aflibercept and Ranibizumab on Hsp60 and Hsp90 Expression in Müller Cells

**Figure 5:**
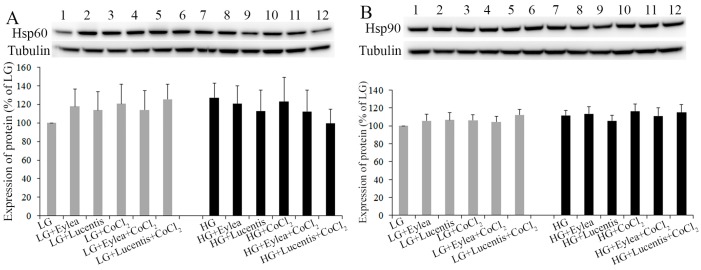
Effects of aflibercept and ranibizumab on Hsp60 and Hsp90 expression in Müller cells under stress conditions. Müller cells were incubated in stress media for 24 h. The effects of aflibercept and ranibizumab on Hsp60 (**A**); and Hsp90 (**B**) expression were studied by Western blots, *n* = 4/group.

In order to study the effects of anti-VEGF therapy on mitochondrial and cytoplasmic stress in Müller cells, we assessed whether ranibizumab and aflibercept affected the expression of Hsp60 and Hsp90 under stress (Figure 5). Hsp70 and Hsp90 are molecular chaperones that are expressed constitutively under normal conditions to maintain protein homeostasis and both are upregulated by environmental stress [24,25]. Hsp60 has primarily been known as a mitochondrial protein that is important for folding key proteins in the mitochondria [25]. Hsp90 interacts with unfolded proteins to prevent irreversible protein aggregation and assists refolding, intracellular transport, maintenance and degradation of proteins in the cytoplasm [24]. We found that hypoxia and HG, either alone or in combination, tended to increase the expression of Hsp60 and Hsp90 when compared with the groups cultured in starvation media containing LG (Figure 5A,B). However, ranibizumab and aflibercept did not have a significant impact on the expression of Hsp60 and Hsp90 in Müller cells under the stress conditions we tested (Figure 5A,B).

### 2.6. Effects of Aflibercept and Ranibizumab on Hsp60 and Hsp90 Expression in Photoreceptors

**Figure 6:**
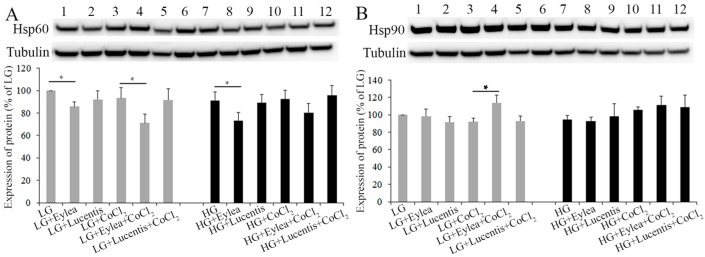
Effects of aflibercept and ranibizumab on Hsp60 and Hsp90 expression in Y79 photoreceptors. Y79 cells were incubated in stress media for 24 h. The effects of aflibercept and ranibizumab on Hsp60 (**A**); and Hsp90 (**B**) were studied by Western blots. * *p* < 0.05, *n* = 6/group.

We also conducted Western blots to study the effects of aflibercept and ranibizumab on expression of Hsp60, Hsp90 in Y79 cells exposed to stress conditions. We found that the addition of aflibercept into culture media significantly inhibited the expression of Hsp60 compared with cells cultured in starvation media containing LG, LG + CoCl_2_ or HG (*p* < 0.05, Figure 6A), and tended to decrease Hsp60 expression in cells cultured in HG + CoCl_2_ (Figure 6A). Aflibercept significantly increased the expression of Hsp90 in cells cultured in LG + CoCl_2_ (Figure 6B). However, ranibizumab did not have a significant effect on the expression of Hsp60 and Hsp90 in Y79 cells under the stress conditions we tested (Figure 6A,B).

### 2.7. Effects of Aflibercept and Ranibizumab on Thioredoxin 1 and 2 (TRX1, TRX2) Expression in Photoreceptors

**Figure 7:**
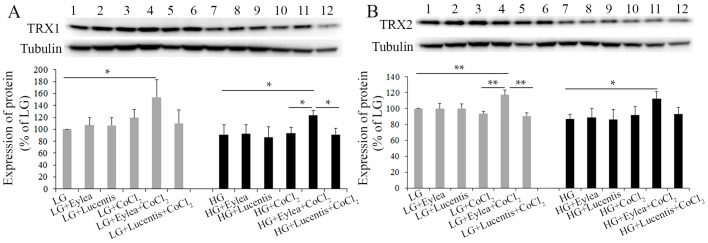
Effects of aflibercept and ranibizumab on expression of TRX1 (**A**) and TRX2 (**B**) in photoreceptor cells. Aflibercept significantly increased the expression of TRX1 and TRX2 under stress conditions caused by CoCl_2_-induced hypoxia. However, ranibizumab had little effects on the expression of TRX1 and TRX2 under the conditions tested. * *p* < 0.05 and ** *p* < 0.01, *n* = 4/group. TRX, thioredoxin.

As our data from Western blots indicated that aflibercept affected the expression of Hsp60 and Hsp90 in Y79 photoreceptors, we next studied the effects of aflibercept and ranibizumab on expression of redox proteins including thioredoxin 1 and 2 (TRX1, TRX2) in Y79 cells. TRX1 is present in the cytosol, while TRX2 is localized to mitochondria [26]. We found that addition of aflibercept into starvation media containing LG + CoCl_2_ or HG + CoCl_2_ significantly increased the expression of TRX1 and TRX2, while ranibizumab did not affect TRX1 and TRX2 expression in Y79 photoreceptors cultured in LG or HG, either with or without CoCl_2_-induced hypoxia (Figure 7).

### 2.8. Effects of Aflibercept and Ranibizumab on Neurotrophic Factors in Müller Cells

**Figure 8:**
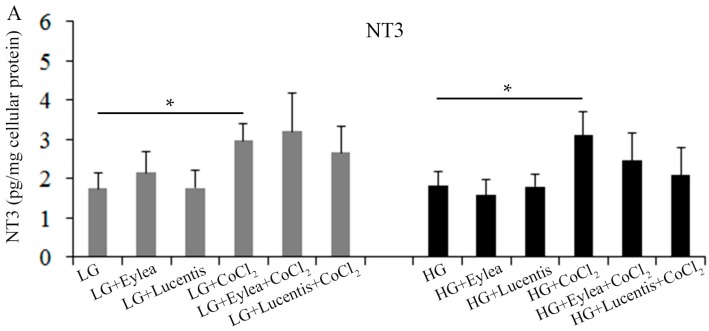

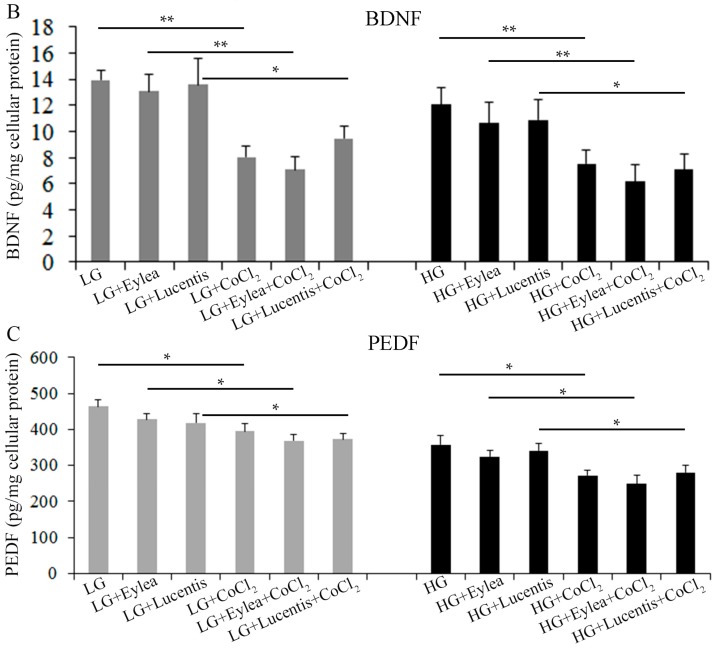
Aflibercept and ranibizumab did not affect the production of NT3, BDNF and PEDF in Müller cells. (**A**) Hypoxia significantly increased NT3 production compared with cells incubated in the medium containing LG or HG; (**B**,**C**) Hypoxia significantly reduced BDNF and PEDF and HG also significantly reduced PEDF production in (**C**). However, the addition of aflibercept and ranibizumab into media did not significantly affect the production of NT3, BDNF and PEDF in (**A**–**C**). * *p* < 0.05 and ** *p* < 0.01, *n* = 9/group in (**A**) and *n* = 6/group in (**B**,**C**). NT3, neurotrophin 3; BDNF, brain-derived neurotrophic factor; PEDF, pigment epithelium derived factor.

As Müller cells play an important role in the maintenance of photoreceptor health through production of neurotrophic factors, we next studied the effects of aflibercept and ranibizumab on the production of NT3, BDGF and PEDF under stress conditions. Compared with cells cultured in the starvation medium containing LG, HG had little effect on NT3 production but CoCl_2_-induced hypoxia significantly increased NT3 production regardless of the concentrations of glucose (Figure 8A). Addition of clinical doses of aflibercept and ranibizumab into the culture media did not have a significant impact on NT3 production in Müller cells cultured in the stress conditions we tested (Figure 8A).

### 2.9. Effects of Aflibercept and Ranibizumab on Interphotoreceptor Retinoid-Binding Protein (IRBP) Production in Y79 Photoreceptors

Measurements of BDNF and PEDF production indicated that hypoxia significantly inhibited the production of BDNF and PEDF in Müller cells cultured in starvation media containing LG or HG (Figure 8B,C). However, neither aflibercept nor ranibizumab affected the levels of BDNF and PEDF production under stress conditions (Figure 8B,C).

**Figure 9:**
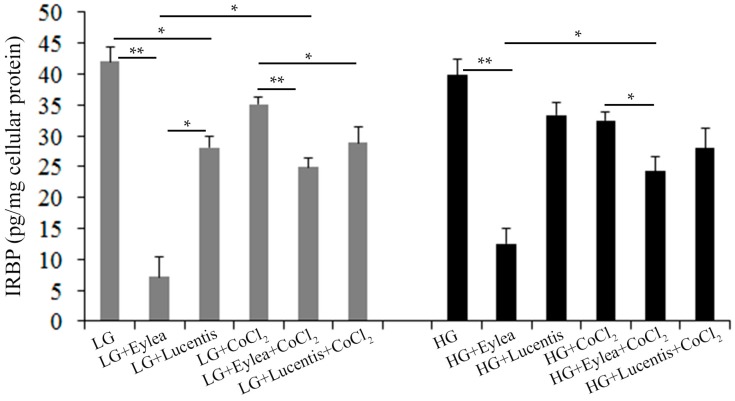
Aflibercept and ranibizumab inhibited IRBP production in Y79 photoreceptor cells. IRBP was measured by ELISA using conditioned media collected from Y79 cells 24 h after incubation in stress media containing 1% FCS. * *p* < 0.05 and ** *p* < 0.01, *n* = 5/group.

As IRBP is expressed by Y79 photoreceptor cells and plays an important role in the visual cycle through transport of 11-*cis* retinal and all-*trans* retinol between the photoreceptors and RPE cells [27,28,29,30,31], we next determined the effects of aflibercept and ranibizumab on the production of IRBP in Y79 photoreceptors (Figure 9). Compared with cells cultured in the starvation medium containing LG, HG slightly reduced the production of IRBP but the differences were not statistically significant (*p* > 0.05, Figure 9). Hypoxia significantly reduced the production of IRBP regardless the concentrations of glucose (*p* < 0.05, LG vs. LG + CoCl_2_ and HG vs. HG + CoCl_2_; Figure 9). Addition of clinical doses of aflibercept and ranibizumab into culture media significantly inhibited the production of IRBP and this inhibitory effect was more profound in cells treated with aflibercept than in those treated with ranibizumab under each stress condition (Figure 9). Interestingly, aflibercept but not ranibizumab, appeared to have less effect on inhibiting IRBP in cells exposed to hypoxia regardless of the concentrations of glucose (Figure 9).

## 3. Discussion

We have studied the potential adverse effects of aflibercept and ranibizumab on human MIO-M1 Müller cells and Y79 photoreceptors under various conditions of stress in vitro. We did not find direct evidence that aflibercept and ranibizumab affected the viability of either type of cells. Neither aflibercept nor ranibizumab affected the production of neurotrophic factors, including NT3, BDNF and PEDF, by Müller cells, nor did they affect the expression of cell stress markers, including Hsp60 and Hsp90. In photoreceptors, however, aflibercept but not ranibizumab, affected the expression of heat shock proteins including Hsp60 and Hsp90 and redox proteins including TRX1 and TRX2. Our most interesting finding was that both aflibercept and ranibizumab inhibited the production of IRBP in Y79 photoreceptors, with the most profound effect observed with aflibercept treatment. Collectively, we found that aflibercept and ranibizumab did not have adverse effects on human Müller cells but their potential influence on photoreceptors should be specifically monitored in clinical research.

Of the various forms of stress we induced, we found that hypoxia was the main factor that drove cell injury and overexpression of VEGF and HIF1α in Müller cells. This observation is consistent with the well-established role of VEGF and HIF1α in mediating neovascularization in ischemic retinal diseases [1,2,32]. VEGF is produced by several types of retinal cells, including Müller cells and RPE cells. Under pathological conditions, exogenous stress leads to morphological, biochemical and physiological changes in Müller cells, including uncontrolled production of VEGF, thus contributing to breakdown of the blood retinal barrier and the development of retinal neovascularization. Müller cells are a major contributor to retinal vascular leakage and pre-retinal and intra-retinal neovascularization in diabetic retinopathy [33].

Y79 photoreceptors have been reported to overexpress VEGF under hypoxic conditions [34]. We confirmed that hypoxia was the main factor that drove cell injury and overexpression of VEGF and HIF1α in Y79 photoreceptors. Photoreceptor cells are the most prevalent cells in the retina. They have a very high metabolic rate and consume more oxygen than other cells throughout the body. There is increasing evidence that photoreceptor cells play a previously unappreciated role in retinal diseases. It has been reported that stressed photoreceptors likely contribute to retinal hypoxia and subsequent retinal abnormalities in early and advanced stages of diabetic retinopathy [35,36,37]. Du et al. [38] reported that photoreceptors were the major source of reactive oxygen species in the retina. Genetic and chemical depletion of photoreceptors inhibited increase in superoxide and inflammatory proteins in diabetic mice [38]. A recent study by Joyal et al. demonstrated that stressed photoreceptors secreted VEGF through stabilizing HIF1α and contributed to deep retinal neovascularization in VLDLR mutant mice [39]. Collectively, these observations indicate that the high energy demand by photoreceptors makes them vulnerable to cell injury under stress conditions such as hypoxia, hyperglycemia and energy starvation, thus acting as both a victim and a contributor to vascular abnormalities in diseased conditions.

We did not find direct evidence that the clinical doses of aflibercept and ranibizumab affected the survival of human MIO-M1 Müller cells and Y79 photoreceptors. This result is consistent with findings reported by others that clinical doses of aflibercept, ranibizumab and bevacizumab did not affect the cell survival of human RPE, rat ganglion cells or mouse 661w photoreceptors [20,21,22,23,40], However, a recent study found that at 10× clinical doses, aflibercept, but not ranibizumab, significantly decreased the cell viability of human RPE [20]. Aflibercept at 2× and 10× clinical doses also caused damage to mitochondria in the RPE [20]. We have studied the effects of clinical doses of aflibercept and ranibizumab on the expression of mitochondrial stress marker Hsp60 and cytoplasmic stress marker Hsp90 along with redox proteins including TRX1 and TRX2 in Müller cells and Y79 photoreceptors. We found that aflibercept and ranibizumab had little direct effect on Müller cells. In photoreceptors, however, aflibercept, but not ranibizumab, caused differential expression of Hsp60, Hsp90; TRX1 and TRX2, indicating that photoreceptors may be more susceptible to aflibercept-induced cellular toxicity.

A number of recent studies have reported the adverse effects of anti-VEGF therapy in vitro and in vivo. Brar et al. reported that VEGF protected retinal ganglion cells against H_2_O_2_-mediated oxidative stress but this protective effect was eliminated by co-treatment with bevacizumab [10]. Pretreatment of the RPE with neutralizing antibodies against VEGF-A promoted H_2_O_2_-induced cell death [8]. In a rat model of ischemia-reperfusion injury, ischemic preconditioning increased the levels of VEGF-A expression and substantially decreased cell apoptosis in ganglion cells and the inner retinal neurons and the neuroprotective effect of ischemic preconditioning was reversed by VEGF-A inhibition [41]. Chronic inhibition of VEGF-A using soluble VEGF-R1 was reported to lead to loss of retinal ganglion cells in normal rats [41]. Klettner et al. reported that aflibercept but not ranibizumab was taken up and stored in the RPE, leading to reduced phagocytic ability and impaired wound healing capacity [42,43]. Considering that photoreceptors do not have the capacity of phagocytosis, the cellular mechanisms underlying aflibercept-induced stress in photoreceptors would be different from that observed in the RPE. Intravitreal injection of bevacizumab disrupted mitochondria in the photoreceptor inner segments and caused a significant increase in photoreceptor apoptosis [44,45]. Sustained neutralization of intraocular VEGF has been reported to induce retinal neurodegeneration in diabetic mice [11]. Januschowski et al. observed significant reduction in a-wave and b-wave amplitudes after short-term exposure of isolated bovine retinas to an oxygen-saturated nutrient solution containing aflibercept [46]. Genetic disruption of VEGF expression in the RPE led to choriocapillary atrophy, RPE and Bruch’s membrane abnormalities, increased photoreceptor apoptosis and reduced cone photoreceptor function [6,7,14]. Collectively, these data suggest the potential adverse effects of anti-VEGF therapy should be carefully evaluated in all types of retinal cells.

One important function of Müller cells is to release neurotrophic factors such as NT3, BDNF and PEDF to maintain the health of photoreceptors and other neurons [18,47,48,49,50]. We next studied the effects of aflibercept and ranibizumab on the production of NT3, BDNF and PEDF in human Müller cells. We found that hypoxia increased NT3 and reduced the production of BDNF and PEDF. However, aflibercept and ranibizumab did not significantly affect the production of these neurotrophic factors by Müller cells under the stress conditions we tested. Since aflibercept and ranibizumab did not affect the cell viability and expression of Hsp60 and Hsp90 in Müller cells, we believe that clinical doses of aflibercept and ranibizumab are not toxic to Müller cells.

Perhaps our most interesting finding was that aflibercept and ranibizumab inhibited the production of IRBP in photoreceptors. IRBP is a major soluble glycoprotein secreted by photoreceptors [51]. It is distributed within the interphotoreceptor matrix (IPM) in a light-dependent manner and participates in the intimate interactions between the IPM and photoreceptors [52]. IRBP is important for the maintenance of the function of photoreceptors through transport of 11-*cis* retinal and all-*trans* retinol between the photoreceptors and RPE cells in the visual cycle, buffering excess vitamin A in the IPM and protecting retinoids from degradation [52]. IRBP deficiency has been linked to photoreceptor damage in a number of retinal diseases including inherited retinal diseases [53] and diabetic retinopathy [31]. We found that clinical doses of aflibercept and ranibizumab significantly inhibited the production of IRBP in Y79 photoreceptors, with the most profound effect observed with aflibercept treatment. Data from Western blots indicated that aflibercept, but not ranibizumab, affected the expression of mitochondrial markers Hsp60 and TRX2 as well as cytoplasmic marker Hsp90 and TRX1 in Y79 cells, suggesting that aflibercept may stress photoreceptors more than ranibizumab does.

Interestingly, we found that aflibercept, but not ranibizumab, had less effect on inhibiting IRBP in hypoxia when compared with cells treated with aflibercept in normoxia. One possible explanation for this observation could be that hypoxia induces overexpression of VEGF-B and PLGF [54] and both angiogenic factors may inhibit the production of IRBP. Given the facts that aflibercept inhibits VEGF-A, -B and PLGF while ranibizumab mainly acts on VEGF-A, aflibercept may partially reverse the inhibitory effects of hypoxia-induced overexpression of VEGF-B and PLGF on IRBP production. Future experiments are warranted to study the effects of different isoforms of VEGF family members on IRBP production in photoreceptors.

We acknowledge that there are limitations in extrapolating findings from in vitro studies to the clinic. Isolation and culture of photoreceptor cells from normal eyes has been a major challenge for retinal research. This is because that normal photoreceptor cells would not survive for very long once deprived of their normal extracellular matrix and cellular contacts with other retinal cells such as the RPE and Müller cells. Since Y79 photoreceptors were originally derived from a human retinoblastoma, they may not act as normal human photoreceptors in vitro in every respect although they express several markers of differentiated photoreceptors including opsin, arrestin, phosducin and IRBP [27,28,29,30,31]. Therefore, our results cannot be readily extrapolated to the clinical setting but may nevertheless be useful to generate hypotheses for testing in clinical trials.

## 4. Materials and Methods

### 4.1. Culture of Human Müller Cells and Photoreceptors under Normal and Stressed Conditions

Human MIO-M1 Müller cells were cultured in DMEM (GIBCO#11885, Grand Island, NY, USA), and retinoblastoma Y79 photoreceptor cells (ATCC^®^ HTB18™, Manassas, VA, USA) were cultured in RPMI1640 (Invitrogen#11875, Sydney, Australia) media containing 10% fetal calf serum (FCS), 1 mg/mL glutamine, 100 U/mL penicillin and 100 U/mL streptomycin. The media were supplemented with various concentrations of glucose to mimic physiologic and hyperglycemic conditions. In order to induce a hypoxic environment, cobalt chloride (CoCl_2_, 200 µM, Sigma, St. Louis, MO, USA) added to media to induce hypoxia-related cell stress [55]. CoCl_2_ establishes the cellular hypoxic response via stabilizing hypoxia-inducible factors through inhibition of the prolyl hydroxylase domain [56,57]. For experiments in which cells were exposed to hypoxic stress, cells were initially cultured in media containing 10% FCS and 5 mM glucose for MIO-M1 cells or 11 mM glucose for Y79 cells and then stressed in “starvation” media containing 1% FCS, various concentrations of glucose, 1% insulin-transferrin-selenium-ethanolamine supplements (ITS-X, GIBCO#51500056), 1 mg/mL glutamine, 100 U/mL penicillin and 100 U/mL streptomycin along with or without CoCl_2_ (200 µM). In order to study the effects of aflibercept (Eyelea) and ranibizumab (Lucentis) on Müller cells and photoreceptors, both types of retinal cells were treated with these two anti-VEGF agents for 24 h under hypoxic stress as described below.

### 4.2. Anti-VEGF Treatments in Human Müller Cells and Photoreceptors

Clinical doses of aflibercept (0.5 mg/mL) and ranibizumab (0.125 mg/mL) were dissolved in starvation media containing 1% FCS, various concentrations of glucose along with and without CoCl_2_ (200 µM) (Table 1). The clinical doses were calculated by assuming that the amount of each drug used clinically in intravitreal injections distributes equally throughout the 4 mL of human vitreous. Müller cells and Y79 photoreceptors were incubated in 12 starvation media containing 1% FCS and other elements including: (1) low glucose (LG); (2) LG + aflibercep; (3) LG + ranibizumab; (4) LG + CoCl_2_ (200 µM; (5) LG + CoCl_2_ + aflibercep; (6) LG + CoCl_2_ + ranibizumab; (7) high glucose (HG), (8) HG + aflibercep; (9) HG + ranibizumab; (10) HG + CoCl_2_; (11) HG + CoCl_2_ + aflibercep; and (12) HG + CoCl_2_ + ranibizumab (Table 1). The effects of aflibercept and ranibizumab on cell survival, differential expression of cell stress markers and the production of NT3, BDNF and PEDF as well as IRBP were assessed 24 h after incubation as described below.

### 4.3. Calcein-AM Cell Viability Assay

Cell viability was measured using the retention of acetoxymethyl ester of calcein (calcein-AM) in live cells as we described previously [58,59]. Calcein-AM is membrane-permeant and can be introduced into cells via incubation with culture media. Once inside the cells, the non-fluorescent calcein-AM is hydrolyzed by intracellular esterases into the highly negatively charged green fluorescent calcein in the cytoplasm of live cells. Calcein-AM cell viability assay was conducted in 48-well plates. For calcein-AM cell viability assays in MIO-M1 Müller cells, 1 × 10^4^ cells were seeded in each well until 80% confluence and then the media were replaced by 200 µL of various stress media and incubated for 24 h. Cell viability assay was conducted by addition of 100 µL of 12 µM calcein-AM (Molecular Probes, Invitrogen) into each well to form a final working concentration of 4 µM calcein-AM. Cells were incubated for 60 min to allow the dye to be taken up. For calcein-AM cell viability assays in Y79 cells, 2 × 10^4^ cells were directly incubated in 200 µL of test media for 24 h and then a volume of 100 µL of 12 µM calcein-AM was added to each well. Fluorescence measurements were performed 60 min after incubation using a Tecan Safire2 fluorescence multi-well plate reader (Tecan, Männedorf, Switzerland) with excitation/emission wavelengths at 485/535 nm. Fluorescent images were also taken using an inverted fluorescent microscope to document the cell morphology after calcein-AM staining.

### 4.4. Measurements of VEGF-A, NT3, BDNF, PEDF and IRBP in Conditioned Media Using Enzyme-Linked Immunosorbent Assays

As FCS contains a complex mixture of growth factors, the production of VEGF-A, NT3, BDNF and PEDF in Müller cells and IRBP in photoreceptors were measured using enzyme-linked immunosorbent assays (ELISA) 24 h after incubating cells in test media containing 1% FCS and various concentrations of glucose along with and without CoCl_2_ (200 µM). In brief, conditioned media were collected from 6-well plates and ELISA was conducted to detect human VEGF-A (R&D Systems, #DY293B, Minneapolis, MN, USA), NT3 (Biosensis, #BEK-2221, Thebarton, Australia), BDNF (R&D Systems, #DY248), PEDF (R&D Systems, #DY1177) and IRBP (LifeSpan Biosciences, #LS-F7906, Seattle, WA, USA) according to the manufacturer’s instructions. Results were normalized to levels of proteins extracted from cells where the conditioned media were collected.

### 4.5. Western Blot Analysis

Western blots were conducted to study changes in hypoxia-inducible factor-α (HIF1α), heat shock proteins (Hsp) 60 and 90 and redox proteins including thioredoxin 1 and 2 (TRX1, TRX2). Briefly, proteins were extracted from MIO-M1 Müller cells and Y79 photoreceptors and their concentrations were determined by bicinchoninic acid (BCA) assays (QuantiPro BCA assay kit, Sigma). Equal amounts of protein were subjected to sodium dodecyl sulfate polyacrylamide gel electrophoresis then transferred to a polyvinylidene difluoride membrane. Membranes were probed with primary antibodies against HIF1α (1:500, Novus Biologicals #NB-100-449, Littleton, CO, USA), Hsp60 (1:1000, Cell Signaling#4870, Beverly, MA, USA), Hsp90 (1:1000, Cell Signaling#4877), TRX1 (1:1000; Cell Signaling #2429) and TRX2 (1:500, Proteintech#13089-1-AP) and then incubated with secondary antibodies conjugated with horseradish peroxidise. Protein bands were visualized using the G:Box BioImaging system (Syngene, Cambridge, UK) and quantified using the GeneTools image scanning and analysis package (Syngene, software version 3.07(g)). Protein expression was normalized to α/β tubulin (1:2000; Cell Signaling #2148), which serves as a loading control.

### 4.6. Statistical Analysis

Differences between study groups were analyzed using one-way analysis of variance (one-way ANOVA) followed by a post hoc Bonferroni’s correction or Kruskal–Wallis tests for multiple comparisons using GraphPad Prism V.5.0 version statistics program (GraphPad Software, San Diego, CA, USA). A *p*-value < 0.05 was considered to be statistically significant. Data are reported as mean ± standard error of the mean (SEM) where applicable.

## 5. Conclusion

We have studied the potential adverse effects of aflibercept and ranibizumab on human Müller cells and retinoblastoma Y79 photoreceptors. Aflibercept and ranibizumab did not appear to affect Müller cells adversely. On the other hand, aflibercept appeared to stress Y79 photoreceptors more compared with ranibizumab. Aflibercept and ranibizumab both inhibited the production of IRBP in Y79 photoreceptors, with the most profound effect observed with aflibercept treatment. Our results indicate that the potential influence of aflibercept and ranibizumab on photoreceptors should be specifically monitored in clinical research.

## Figures and Tables

**Table 1:** Stress conditions in which 1% FCS starvation media were used to test the effects of anti-VEGF therapy on MIO-M1 Müller cells and Y79 photoreceptors.

Group	Glucose (LG/HG)	CoCl_2_ (200 µM)	Aflibercept (0.5 mg/mL)	Ranibizumab (0.125 mg/mL)
1	LG	−	−	−
2	LG	−	+	−
3	LG	−	−	+
4	LG	+	−	−
5	LG	+	+	−
6	LG	+	−	+
7	HG	−	−	−
8	HG	−	+	−
9	HG	−	−	+
10	HG	+	−	−
11	HG	+	+	−
12	HG	+	−	+

LG = low glucose, 5 mM for MIO-M1 Müller cells and 11 mM for Y79 photoreceptors respectively; HG = high glucose, 25 mM for both types of retinal cells. All media were supplemented with 1% insulin-transferrin-selenium-ethanolamine supplements (ITS-X, GIOCO#51500056).
